# State-of-the-art in professional voice

**DOI:** 10.1016/j.bjorl.2021.05.016

**Published:** 2021-10-22

**Authors:** Agricio Crespo, Gustavo Korn

**Affiliations:** a Universidade Estadual de Campinas (UNICAMP), Divisão de Otorrinolaringologia & Cirurgia de Cabeça e Pescoço, Campinas, SP, Brazil; b Universidade Federal de São Paulo (UNIFESP), São Paulo, SP, Brazil

Professional voice is defined as a means of oral communication used by people who depend on it for their occupational activity.^1^ Approximately one third of the professions involve the use of voice professionally.^2^ Voice professionals include, in addition to singers, actors and broadcasters, teachers, lawyers, politicians, telemarketers, and salesclerks, among others.

Particularly in the groups of singers, actors and broadcasters, any vocal alteration may have a very important impact, both in the psychological and economic spheres, since their financial gain depends on the use of the voice.

When caring for a professional voice, it is essential to pay heed to ethical and legal aspects, such as the employees’ attitude at the reception desk, and verify who can accompany the consultation. When talking to the patient, it is vital to be careful with the words and always provide support. Regarding the production team, always remember medical confidentiality. In case of an unfavorable diagnosis, patient privacy must be maintained.^3^ Our country lacks legislation regarding vocal alterations, which are essential, especially for the group of teachers, the subject of several scientific studies.^4^ It is necessary to develop legislation on work-related vocal disorders, similar to the existing one for hearing loss caused by noise exposure.

In the beginning of the COVID-19 pandemic, the Federal Council of Medicine authorized the practice of remote medical assessment, which obviously depends on an adequate internet connection. Despite being widely used by speech therapists, the lack of static and dynamic evaluation of the larynx and vocal tract is a critical point in this practice. Many doubts persist about the safety of professional use of the voice in the pandemic and in the near future, despite many recommendations.^5^ The use of the polymerase-chain reaction (PCR) test for COVID-19 before the presentation, recommendations regarding tests, among others, even when feasible, do not always correspond to the observed reality. It is understood that remote medical assessment in laryngology is a current topic that deserves further discussion.

Voice professionals have been the subject of numerous studies in recent years. However, some points still deserve further study, such as vocal rest recommendations (either relative or absolute) and the use of corticosteroids. It is important to recall that the study of voice professionals shows many variables, when compared, for instance, to a population with a specific larynx disorder, such as focal laryngeal dystonia.

One may say that the state-of-the-art when caring for a voice professional can be defined as embracing the professional approach, performing a careful anamnesis, focused on the specificity of their complaint; consider psychological and emotional aspects (especially during this pandemic period); perform a voice evaluation, complete otorhinolaryngological examination, as well as static and dynamic evaluation of the larynx and vocal tract. The propaedeutics includes nasofibrolaryngoscopy, laryngoscopy and stroboscopy. The functional assessment, during which the patient can “demonstrate” exactly where their complaint, problem or limitation is, is always crucial. The multiprofessional team includes a speech therapist, singing teacher, musical director and musical coach, among others.

## Conflicts of interest

The authors declare no conflicts of interest.
